# Effects of Resveratrol on Browning and Insulin Signaling in Primary Murine Adipocytes: Modulation by Sex and Diabetic Status

**DOI:** 10.3390/nu18010019

**Published:** 2025-12-19

**Authors:** Xinyun Xu, Haoying Wu, Jiangang Chen, Shu Wang, Ling Zhao

**Affiliations:** 1 Department of Nutrition, University of Tennessee, Knoxville, TN 37996, USA; xxu28@vols.utk.edu (X.X.); hwu26@vols.utk.edu (H.W.); 2 Department of Public Health, University of Tennessee, Knoxville, TN 37996, USA; jchen38@utk.edu; 3 College of Health Solutions, Arizona State University, Phoenix, AZ 85004, USA; shu.wang.10@asu.edu

**Keywords:** resveratrol, stromal cells, sex, diabetes, beige adipocyte, browning

## Abstract

**Background:** Excess accumulation of white adipose tissue is linked to the development of obesity and type 2 diabetes, both of which are associated with systemic metabolic dysfunction. One promising approach is to convert white adipocytes into beige adipocytes, which have greater thermogenic potential and improved insulin sensitivity. Trans-resveratrol (RES), a polyphenolic compound known to have multiple metabolic benefits, has been reported to promote browning of adipocytes and improve insulin signaling; however, it is unclear whether sex and diabetic status modify RES’s effects. **Methods:** We evaluated the ability of RES to induce browning and increase insulin sensitivity in adipose-derived stromal cells (ADSCs) derived from diabetic *db*/*db* mice and explored the extent to which these responses are modulated by sex and diabetic status. Subcutaneous ADSCs were isolated from wildtype (WT) and diabetic (*db*/*db*) male and female mice and then treated with RES during beige adipocyte differentiation. **Results:** RES enhanced the expression of *Pgc1α* and *Ucp1* mRNA and increased mitochondrial proton leak in ADSCs of both WT and *db*/*db* mice. RES also enhanced insulin-induced AKT phosphorylation in all groups of ADSCs. Notably, the effects of RES on browning and insulin signaling were influenced by the sex and diabetic status of the mice, as ADSCs from female diabetic mice responded differently compared with those from their WT or male counterparts. **Conclusions:** These findings highlight the importance of considering sex and diabetic status when developing browning strategies to target obesity and type 2 diabetes.

## 1. Introduction

Obesity and its associated metabolic diseases, primarily type 2 diabetes (T2DM), have become a major concern for public health globally [1,2]. Excessive accumulation of body fat, particularly white adipose tissue (WAT) [3], contributes to metabolic disorders. This occurs at least in part through its role in chronic inflammation [4] and insulin resistance [5]. One promising therapeutic strategy to combat excess WAT is “browning”, i.e., the induction of beige adipocytes within WAT depots [6,7], resulting in increased mitochondria numbers and increased UCP1 expression, similarly to classical brown fat [8,9,10]. WAT browning increases fatty acid and glucose uptake and utilization, which increases energy expenditure (via heat production) and insulin sensitivity throughout the body [8,9,10].

Resveratrol, a polyphenolic compound found most abundantly in grapes and red wine, has been shown to positively impact metabolic health [11,12]. Many studies have demonstrated that RES decreases fat accumulation and insulin resistance, and also induces browning of subcutaneous WAT in vivo and browning of white-adipose-derived stromal cells in vitro [13,14,15,16,17,18,19,20]. As a major cell type in the adipose tissue, adipose-derived stromal cells are stem cell-like and have the potential to differentiate into adipocytes upon stimulation. Studies have shown sex differences in the response of adipose tissue to obesity and aging, with females generally displaying more adaptability [21,22]. Moreover, there were sex differences in browning of white adipose tissue in response to CL316,243, a specific beta-3 adrenergic receptor agonist [23]. However, most studies on the effects of RES on browning have only examined animals of one sex (typically males) in vivo [13,14,15,18,19,20] or stromal cells derived from a single sex in vitro [15]. It is not known whether sex can modulate RES’s effects on browning. Moreover, diabetic status impairs the proliferation, migration, and therapeutic potential of adipose-derived stromal cells for wound healing [24]. However, whether diabetes status affects stromal cells’ browning capacity in response to RES remains unknown. Therefore, the aim of this study is to determine whether sex or diabetic status modulates browning of adipose-derived stromal cells by RES.

In this study, we investigate the modulation of sex and diabetic status on the browning and improvement of insulin sensitivity by RES using white-adipose-derived stromal cells from *db*/*db* and wildtype (WT) mice of both sexes. Understanding the modulation of sex and diabetes status on RES’s browning effects is crucial for precision nutrition in the management of obesity and T2DM.

## 2. Materials and Methods

### 2.1. Reagents

Trans-resveratrol (RES, Batch # 0584598-2, Purity: 100%), indomethacin, and rosiglitazone (ROSI) were purchased from Cayman Chemical (Ann Arbor, MI, USA). Dexamethasone (Dex), 3-isobutyl-L-methylxanthine (IBMX), insulin, 3,3′,5-triiodo-L thyronine (T_3_), and isoproterenol (ISO) were purchased from Millipore Sigma (St. Louis, MO, USA).

### 2.2. Animals

A heterozygous breeding pair of leptin-receptor-deficient mice (B6.BKS(D)-Leprdb/J (Strain#:000697) was purchased from the Jackson Laboratory (Bar Harbor, ME, USA) at six weeks of age and used to generate WT and *db*/*db* littermates in our animal facility. Offspring were genotyped at weaning (3 weeks old) to select WT and *db*/*db* mice for experiments, as previously reported [25]. Mice were housed in a temperature-controlled facility (22 ± 3.6 °C) with a 12 h light/dark cycle and provided ad libitum access to a standard chow diet and water. All experimental procedures were approved by the University of Tennessee’s Institutional Animal Care and Use Committee (IACUC) (Protocol# 2320, 23 December 2023).

### 2.3. Isolation and Differentiation of Adipose-Derived Stromal Cells (ADSCs)

ADSCs were isolated from the inguinal subcutaneous WAT (sWAT) of male and female WT and *db*/*db* mice at 10–12 weeks of age, as described in [26]. Briefly, under anesthesia with isoflurane, mice underwent a terminal blood draw via cardiac puncture, followed by euthanasia with carbon dioxide. The sWAT depots were aseptically dissected and rinsed twice with ice-cold PBS. The tissue was minced, then digested with 55 µL/mL of 100 mg/mL collagenase type I (Worthington Biochemical Corporation, Lakewood, NJ, USA) at 2 mL/mg in isolation buffer at 37 °C for 45 min with gentle shaking. The digested tissue was filtered through a 100 μm cell strainer (Fisher Scientific, Hampton, NH, USA). The filtrate was centrifuged at 500× *g* for 6 min at 4 °C. The stromal vascular fraction was resuspended in Dulbecco’s Modified Eagle Medium (DMEM)/high glucose supplemented with 10% fetal bovine serum (FBS) and 1% penicillin and streptomycin (P/S) and was cultured in a 37 °C incubator with 5% CO_2_. The adherent stromal cells from 3 to 5 mice of the same sex and genotype (*n* = 3–5) were pooled and used for the subsequent experiments.

Brown-like adipocyte differentiation was induced with an induction medium containing DMEM/high glucose with 20% FBS, 5 µg/mL insulin, 1 µM Dex, 0.5 mM IBMX, 125 µM indomethacin, and 1 nM T_3_ for three days. This was followed by four days in a maintenance medium (DMEM/high glucose with 10% FBS, 5 µg/mL insulin, and 1 nM T_3_), as described in [27]. Throughout the differentiation period, the following treatments were applied: (1) no additives (the negative control), (2) DMSO (0.1%) (the vehicle control), (3) RES (10 μM), and (4) ROSI (1 μM). Each treatment was performed in biological triplicate for each genotype and sex. On day 7, cells were treated for 6 h with either isoproterenol (ISO, a non-selective β-adrenergic agonist, 1 μM) to activate brown-like adipocytes (ISO-stimulated conditions) or the vehicle control (H_2_O) (the basal conditions) prior to total RNA isolation.

### 2.4. Semi-Quantitative Real-Time PCR

Total RNA was extracted via TRI Reagent (Molecular Research Center, Inc., Cincinnati, OH, USA). Quantification and quality evaluation of the RNA were performed on a NanoDrop spectrophotometer (Thermo Fisher Scientific, Waltham, MA, USA). A High-Capacity cDNA Reverse Transcription Kit (Applied Biosystems, Foster City, CA, USA) was used for reverse transcription of cDNA from 2 µg of total RNA. The expression levels of brown marker genes *Pgc1α* and *Ucp1* were quantified using SYBR Green PCR Master Mix (Applied Biosystems, Foster City, CA, USA) on a 7300 Real-Time PCR System (Applied Biosystems, Foster City, CA, USA). The 2^−ΔΔCt^ method was used to calculate the relative expression of target genes normalized by the housekeeping gene 36B4, and the results are presented as folds of the negative control group.

### 2.5. Mitochondrial Stress Test

Mitochondrial stress tests were performed on day 8 of differentiation using an XFe24 Analyzer (Agilent Technologies, Santa Clara, CA, USA). Cells were plated in 24-well microplates for at least three hours before the test. The cells were washed with PBS buffer and replaced with Seahorse XF assay medium containing 10 mM glucose, 2 mM glutamine, and 1 mM sodium pyruvate. The cells were then placed in a 37 °C non-CO_2_ incubator for one hour. Oxygen consumption rates (OCR) were recorded at baseline and after each injection: oligomycin (1.5 μM), FCCP (6.5 μM), and rotenone/antimycin A (1 μM each). Basal respiration, ATP-linked respiration, maximal respiration, and non-mitochondrial respiration were calculated following the manufacturer’s instructions.

### 2.6. Western Blot Analysis of Insulin Signaling

Differentiated adipocytes were serum-starved for 15–16 h, followed by 50 nM insulin stimulation for 20 min at 37 °C. The cells were then lysed in RIPA buffer (Cell Signaling Technology, Danvers, MA, USA) containing protease and phosphatase inhibitor cocktails. Protein concentration was determined using the Pierce™ BCA Protein Assay Kit (Thermo Fisher Scientific). Equal amounts of protein were loaded onto SDS-PAGE gels and transferred to PVDF membranes. Membranes were blocked with 5% non-fat milk, followed by incubation with primary antibodies anti-phospho-AKT Ser473 (1:1000; Cell Signaling Technology; Cat# 4060S), anti-(total) AKT (1:1000; Cell Signaling Technology; Cat# 9272), anti-ERK1/2 (1:1000; Cell Signaling Technology; Cat# 4695S), or anti-ERK1/2 (1:1000; Cell Signaling Technology; Cat# 9102S) at 4 °C overnight. ERK1/2 was used as a loading control. Then, membranes were washed and incubated with HRP-conjugated secondary antibodies (1:4000; Cell Signaling Technology; Cat# 7074S) for 1 h at room temperature. Protein bands were detected using the chemiluminescent substrate SuperSignal™ West Pico PLUS (Thermo Fisher Scientific, Waltham, MA, USA) and imaged with the ChemiDoc XRS+ system (Bio-Rad, Hercules, CA, USA). Band intensity was quantified using ImageJ 1.54g (NIH, Bethesda, MD, USA), and AKT phosphorylation was calculated as the ratio of *p*-AKT to total AKT (*p*-AKT/AKT).

### 2.7. Statistical Analysis

Data are presented as mean ± SEM. To evaluate the effects of isoproterenol or insulin compared to the vehicle control, two-way ANOVA followed by Tukey’s post hoc test was performed, where applicable. Within each group, one-way ANOVA was used to compare fold changes relative to the controls. A *p*-value < 0.05 was considered statistically significant. All statistical analyses were conducted using GraphPad Prism 9 (Dotmatics, Inc., San Diego, CA, USA).

## 3. Results

### 3.1. RES Induces Browning by Increasing Pgc1α and Ucp1 mRNA Expression in the Beige Adipocytes

To investigate the browning effect of RES on subcutaneous white-adipose-tissue-derived stromal cells (ADSCs), we carried out brown-like differentiation of the ADSCs for 7 days in the presence or absence of 10 µM of RES, ROSI (as a positive control), or the vehicle control (DMSO). This dose of RES was used because it is considered to be the highest physiological achievable dose [28]. We then assessed mRNA expression of thermogenic markers *Pgc1α* and *Ucp1* under both basal and ISO-stimulated conditions.

**Figure 1:**
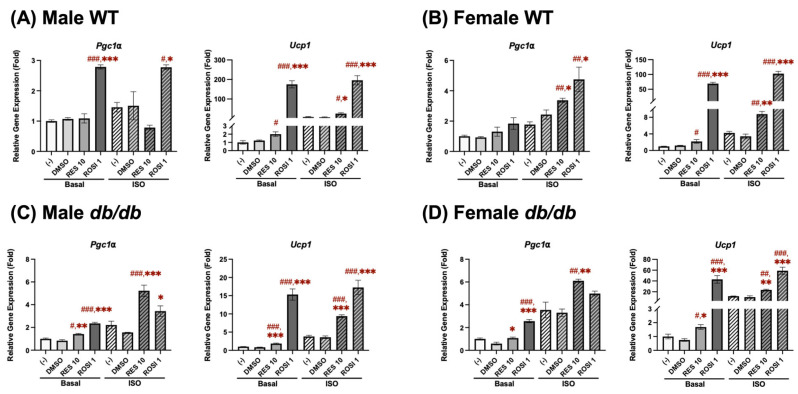
RES induced browning by increasing *Pgc1α* and *Ucp1* mRNA expression in the differentiated ADSCs derived from (**A**) male WT, (**B**) female WT, (**C**) male *db/db*, and (**D**) female *db/db* mice. The ADSCs were isolated from subcutaneous white adipose tissue in mice aged 10–12 weeks. *Pgc1α* and *Ucp1* mRNA expression was measured in pooled ADSCs from 3 to 5 mice following 7-day differentiation in the presence or absence of either resveratrol (RES, 10 µM), rosiglitazone (ROSI, a positive control, 1 µM), or the vehicle control (DMSO). Isoproterenol (ISO, 1 µM) was added for the final 6 h to activate brown-like adipocytes. Data are shown as mean ± SEM (*n* = 3). One-way ANOVA was used for all statistical analyses in basal or ISO-stimulated conditions. ^#^, ^##^, and ^###^ indicate *p* < 0.05, *p* < 0.01, and *p* < 0.001, respectively, compared to the negative control (−) group, while *, **, and *** indicate *p* < 0.05, *p* < 0.01, and *p* < 0.001, respectively, compared to the DMSO group.

In the differentiated male WT ADSCs (Figure 1A), ROSI significantly increased in *Pgc1α* mRNA expression, while no significant changes in *Pgc1α* expression were found in the RES-treated groups compared to the negative and vehicle (DMSO) controls under both basal and ISO-stimulated conditions. For *Ucp1* expression, ROSI and RES robustly increased *Ucp1* mRNA in both basal and ISO-stimulated conditions.

In the differentiated female WT ADSCs (Figure 1B), ROSI and RES both significantly enhanced *Pgc1α* mRNA expression under ISO stimulation, but not under basal conditions, compared to the control groups. In contrast, *Ucp1* mRNA expression was significantly upregulated by ROSI and RES under both basal and ISO-stimulated conditions.

In contrast, a robust increase in *Pgc1α* mRNA expression was shown in the differentiated male *db/db* ADSCs (Figure 1C) following ROSI and RES treatments under both basal and ISO-stimulated conditions. *Ucp1* mRNA expression was similarly elevated by ROSI and RES. A similar pattern was observed in the differentiated female *db/db* ADSCs (Figure 1D). ROSI greatly induced *Pgc1α* mRNA expression compared to RES under basal conditions, but not after ISO stimulation. ROSI also significantly elevated *Ucp1* mRNA expression under both conditions. RES significantly increased both *Pgc1α* and *Ucp1* mRNA expression under both basal and ISO-stimulated conditions.

### 3.2. Modulation of Sex and Diabetes Status on Pgc1α and Ucp1 mRNA Expression in Response to ISO and RES

**Figure 2:**
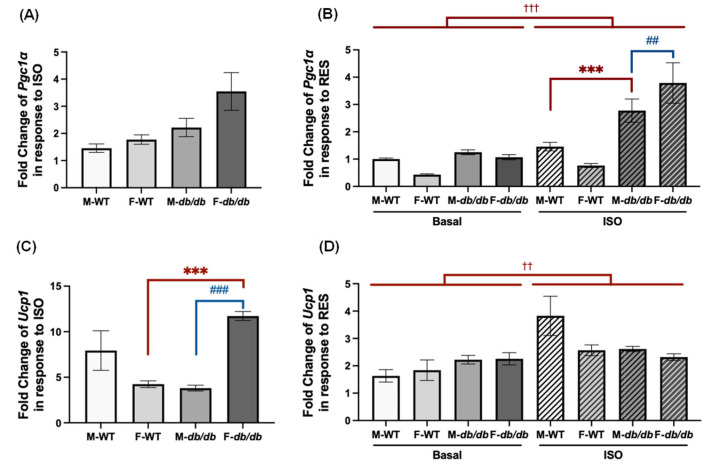
Fold changes in thermogenic gene expression following ISO and RES treatment in the adipocytes derived from ADSCs of male and female WT and *db*/*db* mice. (**A**) Fold Changes in Pgc1α and (**C**) *Ucp1* mRNA expression in response to ISO stimulation were calculated for all groups. (**B**) Fold Changes in Pgc1α and (**D**) *Ucp1* mRNA expression in response to RES relative to the DMSO were calculated under basal or ISO-stimulated conditions. Data are shown as mean ± SEM (*n* = 3). One-way ANOVA was used for statistical analysis in (**A**,**C**); two-way ANOVA was used in (**B**,**D**). ^##^, ^###^ indicate *p* < 0.01 and *p* < 0.001 for comparisons between males and females. *** indicates *p* < 0.001 for comparisons between WT and *db*/*db* mice. ^††^, ^†††^ indicate *p* < 0.01 and *p* < 0.001 for comparisons between basal and ISO conditions.

To understand the impact of genotype and sex on the differentiated ADSCs’ response to ISO and RES, we compared the relative fold changes in *Pgc1α* and *Ucp1* mRNA expression in response to ISO stimulation (Figure 2A,C). No statistically significant differences were found in ISO-stimulated *Pgc1α* upregulation among the cells from male and female WT or *db*/*db* mice (Figure 2A). We further compared the relative fold changes in *Pgc1α* mRNA expression following RES under basal and ISO-stimulated conditions (Figure 2B). RES-induced *Pgc1α* upregulation was higher in the *db*/*db* adipocytes than the WT adipocytes under ISO-stimulated conditions, reaching statistical significance only in male *db/db* adipocytes (Figure 2B). Additionally, male *db*/*db* adipocytes showed higher RES-induced *Pgc1α* upregulation than their female counterparts (Figure 2B).

ISO stimulation induced significant increases in *Ucp1* mRNA expression in the differentiated ADSCs from female *db*/*db* mice compared to female WT mice (Figure 2C). Moreover, differentiated female *db*/*db* ADSCs exhibited significantly greater *Ucp1* induction than their male *db*/*db* counterparts after ISO stimulation (Figure 2C). In comparison, RES induced *Ucp1* upregulation to a similar extent in all groups under both basal and ISO-stimulated conditions, with no significant differences across sexes or genotypes (Figure 2D).

### 3.3. RES Increased Mitochondrial Uncoupling in the Beige Adipocytes

**Figure 3:**
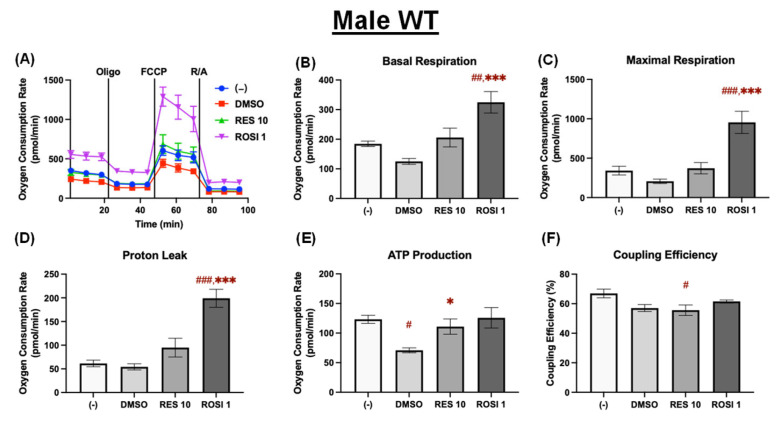
RES enhanced mitochondrial uncoupling in the differentiated ADSCs from male WT mice. The ADSCs were first differentiated in the presence or absence of RES (10 µM), ROSI (1 µM), or DMSO for 7 days, then were reseeded into a Seahorse XFe assay plate. The cells underwent real-time oxygen consumption rate (OCR) measurements for a mitochondrial stress test after reseeding. (**A**) OCR recordings over time, (**B**) basal respiration, (**C**) maximal respiration, (**D**) proton leak, (**E**) ATP production, and (**F**) coupling efficiency are shown. Data are shown as mean ± SEM (*n* = 3–5). One-way ANOVA was used for statistical analysis. ^#^, ^##^, and ^###^ indicate *p* < 0.05, *p* < 0.01, and *p* < 0.001, respectively, compared to the (−) group, while *, *** indicate *p* < 0.05 and *p* < 0.001 compared to the DMSO group.

The mitochondrial stress test was performed to assess mitochondrial respiration in the ADSCs differentiated in the presence or absence of 10 μM of RES, ROSI, or the vehicle control (DMSO). In the differentiated male WT ADSCs, RES did not significantly alter basal respiration, maximal respiration, or proton leak compared to both the (−) and DMSO groups (Figure 3A–D). However, ATP production was significantly increased by RES relative to DMSO, which itself suppressed ATP production compared to the (−) control (Figure 3E). RES further reduced coupling efficiency compared to the (−) group (Figure 3F), indicating enhanced mitochondrial uncoupling, a marker for thermogenic activation. In contrast, ROSI did not change uncoupling efficiency (Figure 3F).

**Figure 4:**
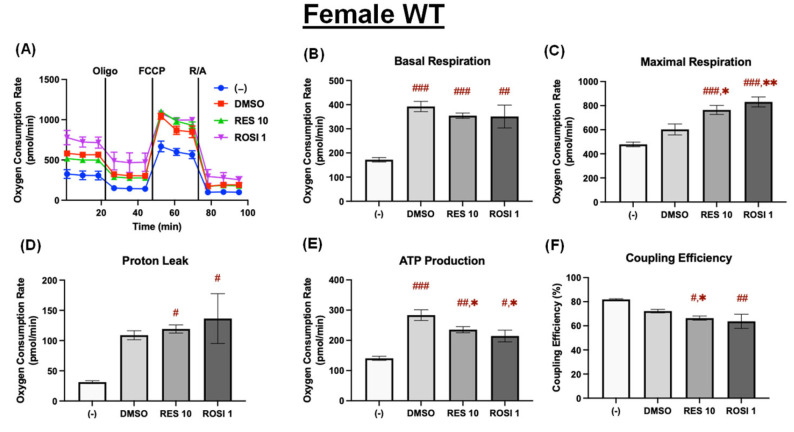
RES enhanced mitochondrial uncoupling in the differentiated ADSCs from female WT mice. The ADSCs were first differentiated in the presence or absence of RES (10 µM), ROSI (1 µM), or DMSO for 7 days, then were reseeded into a Seahorse XFe assay plate. The cells underwent real-time oxygen consumption rate (OCR) measurements for a mitochondrial stress test after reseeding. (**A**) OCR recordings over time, (**B**) basal respiration, (**C**) maximal respiration, (**D**) proton leak, (**E**) ATP production, and (**F**) coupling efficiency are shown. Data are shown as mean ± SEM (*n* = 3–5). One-way ANOVA was used for statistical analysis. ^#^, ^##^, and ^###^ indicate *p* < 0.05, *p* < 0.01, and *p* < 0.001, respectively, compared to the (−) group, while *, ** indicate *p* < 0.05 and *p* < 0.01 compared to the DMSO group.

In the differentiated female WT ADSCs, basal respiration was significantly increased by DMSO, RES, and ROSI compared to the (−) control, with no significant differences among the three treatments (Figure 4A,B). Maximal respiration was significantly elevated by both RES and ROSI compared to the (−) and DMSO controls (Figure 4C), indicating enhanced respiratory capacity. Mitochondrial proton leak was also increased by RES and ROSI relative to the (−) control but not significantly different from the DMSO control (Figure 4D). While both RES and ROSI elevated ATP production compared to the (−) control, both treatments induced modest yet significant reductions in ATP production compared to DMSO (Figure 4E). Finally, RES significantly decreased coupling efficiency compared to both the (−) and DMSO controls, while ROSI had modest effects on coupling efficiency, reducing it significantly only in comparison to the (−) control (Figure 4F).

**Figure 5:**
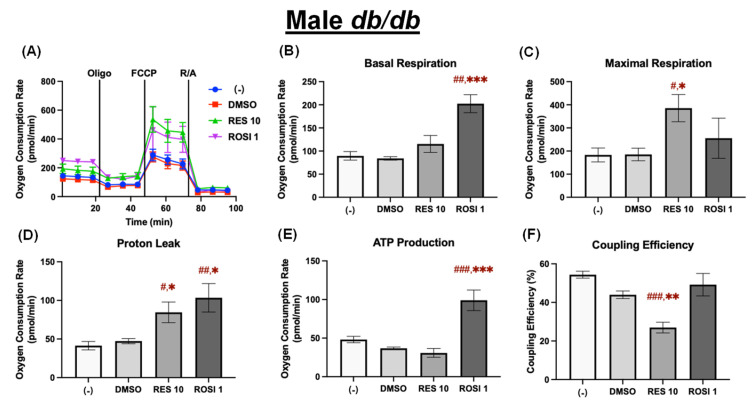
RES enhanced mitochondrial uncoupling in the differentiated ADSCs from male *db*/*db* mice. The ADSCs were first differentiated in the presence or absence of RES (10 µM), ROSI (1 µM), or DMSO for 7 days, then were reseeded into a Seahorse XFe assay plate. The cells underwent real-time oxygen consumption rate (OCR) measurements for a mitochondrial stress test after reseeding. (**A**) OCR recordings over time, (**B**) basal respiration, (**C**) maximal respiration, (**D**) proton leak, (**E**) ATP production, and (**F**) coupling efficiency are shown. Data are shown as mean ± SEM (*n* = 3–5). One-way ANOVA was used for statistical analysis. ^#^, ^##^, and ^###^ indicate *p* < 0.05, *p* < 0.01, and *p* < 0.001, respectively, compared to the (−) group, while *, **, and *** indicate *p* < 0.05, *p* < 0.01, and *p* < 0.001, respectively, compared to the DMSO group.

Similarly to male WT ADSCs, in the differentiated male *db*/*db* ADSCs, ROSI, but not RES, significantly increased basal respiration compared to both the (−) and DMSO controls (Figure 5A,B). In contrast, RES, but not ROSI, significantly increased maximal respiration compared to both controls (Figure 5C). Both RES and ROSI significantly elevated proton leak compared to both controls (Figure 5D). Moreover, ROSI, but not RES, also significantly increased ATP production (Figure 5E). As a consequence, RES significantly decreased coupling efficiency compared to both the (−) and DMSO controls. In contrast, ROSI had no effects on the coupling efficiency in the differentiated male *db*/*db* ADSCs (Figure 5F).

**Figure 6:**
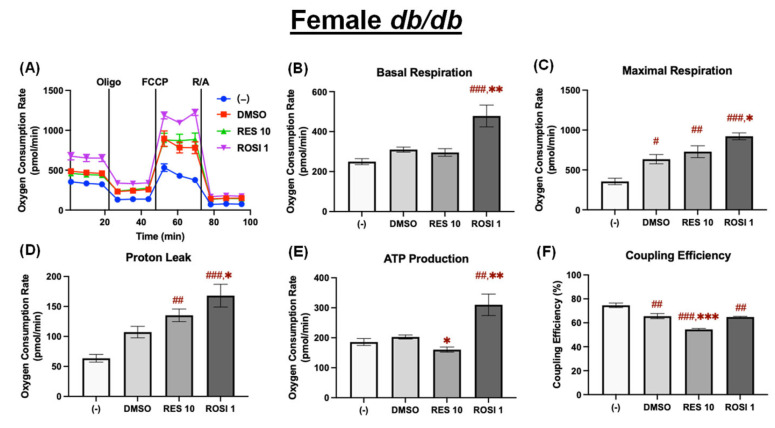
RES enhanced mitochondrial uncoupling in the differentiated ADSCs from female *db/db* mice. The ADSCs were first differentiated in the presence or absence of RES (10 µM), ROSI (1 µM), or DMSO for 7 days, then were reseeded into a Seahorse XFe assay plate. The cells underwent real-time oxygen consumption rate (OCR) measurements for a mitochondrial stress test after reseeding. (**A**) OCR recordings over time, (**B**) basal respiration, (**C**) maximal respiration, (**D**) proton leak, (**E**) ATP production, and (**F**) coupling efficiency are shown. Data are shown as mean ± SEM (*n* = 3–5). One-way ANOVA was used for statistical analysis. ^#^, ^##^, and ^###^ indicate *p* < 0.05, *p* < 0.01, and *p* < 0.001, respectively, compared to the (−) group, while *, **, and *** indicate *p* < 0.05, *p* < 0.01, and *p* < 0.001, respectively, compared to the DMSO group.

In the differentiated female *db*/*db* ADSCs, ROSI, but not RES, significantly increased basal respiration compared to both the (−) and DMSO controls (Figure 6B). Maximal respiration was elevated by DMSO, RES, and ROSI compared to the (−) control (Figure 6C). ROSI significantly increased proton leak and ATP production compared to both the (−) and DMSO controls. In contrast, RES caused a modest but non-significant elevation in proton leak and significantly decreased ATP production compared to the DMSO control (Figure 6D,E). As a result, RES significantly reduced coupling efficiency compared to both controls. In contrast, ROSI had modest effects on coupling efficiency, significantly reducing it only compared to the (−) control in differentiated female *db*/*db* ADSCs (Figure 6F).

### 3.4. Modulation of Sex and Diabetes Status on Mitochondrial Uncoupling in Response to RES in the Beige Adipocytes

**Figure 7:**
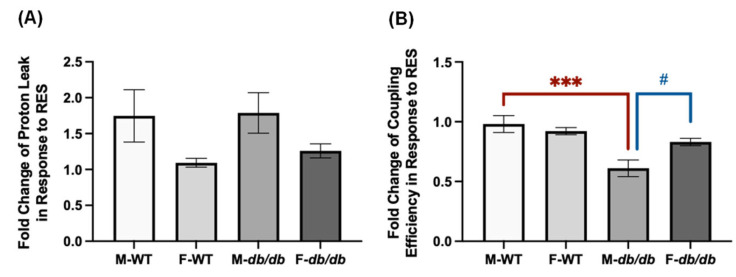
Fold changes in proton leak and coupling efficiency following RES treatment in the differentiated ADSCs from male and female WT and *db*/*db* mice. (**A**) Fold changes in proton leak and (**B**) coupling efficiency in response to RES relative to DMSO were calculated for each of the 4 groups, highlighting sex-specific responses and differences between the WT and *db*/*db*. Data are shown as mean ± SEM (*n* = 3–5). One-way ANOVA was used for statistical analysis. ^#^ indicates *p* < 0.05 in the comparison between sexes, and *** indicates *p* < 0.001 in the comparison between the WT and *db*/*db* mice.

To assess the relative uncoupling effects across different differentiated ADSCs, fold changes in proton leak and coupling efficiency induced by RES relative to the DMSO control were calculated (Figure 7). There were no significant differences among the four groups in the fold changes in proton leak induced by RES compared to the DMSO control (Figure 7A). However, RES reduced coupling efficiency more in differentiated male *db*/*db* ADSCs than in their male WT and female *db*/*db* counterparts (Figure 7B).

### 3.5. RES Improved Insulin Signaling in the Beige Adipocytes

**Figure 8:**
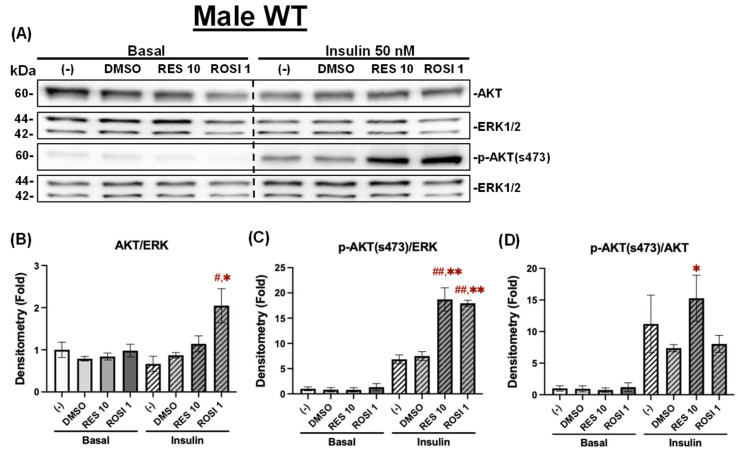
RES improved insulin signaling in the differentiated ADSCs from male WT mice. The ADSCs from the male WT mice were differentiated for 7 days in the presence or absence of RES (10 µM), ROSI (a positive control, 1 μM), or DMSO, followed by overnight starvation in DMEM/high glucose without FBS. Cells were then stimulated with or without insulin (50 nM) for 20 min, and protein lysates were collected for Western blot analysis. (**A**) Total AKT, *p*-AKT (ser473), and their respective loading control ERK1/2 are shown. The left panel corresponds to the basal condition (−Insulin), and the right panel to the insulin-stimulated condition (+Insulin). Quantifications of total AKT/ERK1/2 (**B**), *p*-AKT/ERK1/2 (**C**), and *p*-AKT/total AKT (**D**) are presented as fold changes relative to the (−) group under basal conditions. Data are shown as mean ± SEM (*n* = 3). One-way ANOVA was used for statistical analysis. ^#^ and ^##^ indicate *p* < 0.05 and *p* < 0.01 compared to the (−) group. * and ** indicate *p* < 0.05 and *p* < 0.01 compared to the DMSO group.

Beige adipocytes generated from browning are known to increase glucose uptake and improve systemic glucose metabolism [29]. Given the distinct differences in browning in response to RES, we then assessed and compared RES’s effects on insulin signaling. Specifically, we examined insulin-stimulated AKT phosphorylation on Ser473 in the four groups of ADSCs differentiated in the presence or absence of RES, ROSI, or DMSO. In the differentiated male WT ADSCs, ROSI significantly increased total AKT protein levels only following insulin stimulation compared to both the negative and vehicle controls, whereas RES had no effect on total AKT levels under either basal or insulin-stimulated conditions (Figure 8A,B). Both RES and ROSI significantly increased insulin-stimulated *p*-AKT (Ser473) levels compared to the (−) and DMSO controls (Figure 8C). Consequently, only RES significantly increased the *p*-AKT/AKT ratio compared to DMSO in the differentiated male WT ADSCs (Figure 8D).

**Figure 9:**
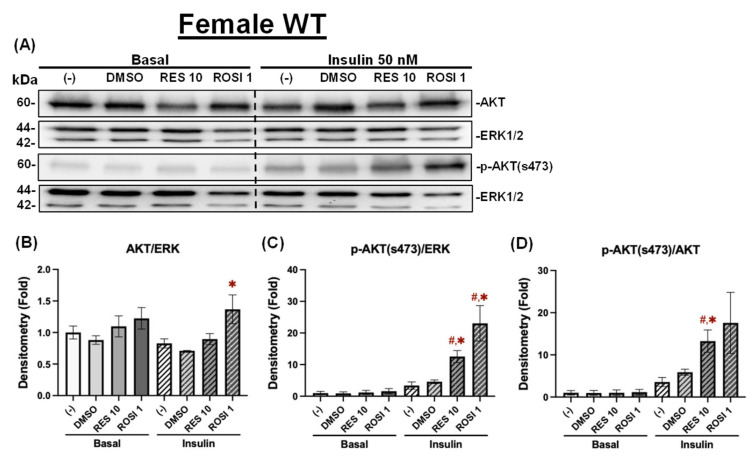
RES improved insulin signaling in the differentiated ADSCs from female WT mice. The ADSCs from the female WT mice were differentiated for 7 days in the presence or absence of RES (10 µM), ROSI (a positive control, 1 μM), or DMSO, followed by overnight starvation in DMEM/high glucose without FBS. Cells were then stimulated with or without insulin (50 nM) for 20 min, and protein lysates were collected for Western blot analysis. (**A**) Total AKT, *p*-AKT (ser473), and their respective loading control ERK1/2 are shown. The left panel corresponds to the basal condition (−Insulin), and the right panel to the insulin-stimulated condition (+Insulin). Quantifications of total AKT/ERK1/2 (**B**), *p*-AKT/ERK1/2 (**C**), and *p*-AKT/total AKT (**D**) are presented as fold changes relative to the (−) group under basal conditions. Data are shown as mean ± SEM (*n* = 3). One-way ANOVA was used for statistical analysis. ^#^ indicates *p* < 0.05 compared to the (−) group. * indicates *p* < 0.05 compared to the DMSO group.

Similar results were found in the differentiated female WT ADSCs. ROSI, but not RES, significantly increased total AKT levels following insulin stimulation compared to DMSO (Figure 9A,B). Under insulin stimulation, both RES and ROSI significantly elevated *p*-AKT (Ser473) levels compared to the (−) and DMSO controls (Figure 9C). While both treatments increased the *p*-AKT/AKT ratios, only RES’s effects reached statistical significance compared to both controls (Figure 9D).

**Figure 10:**
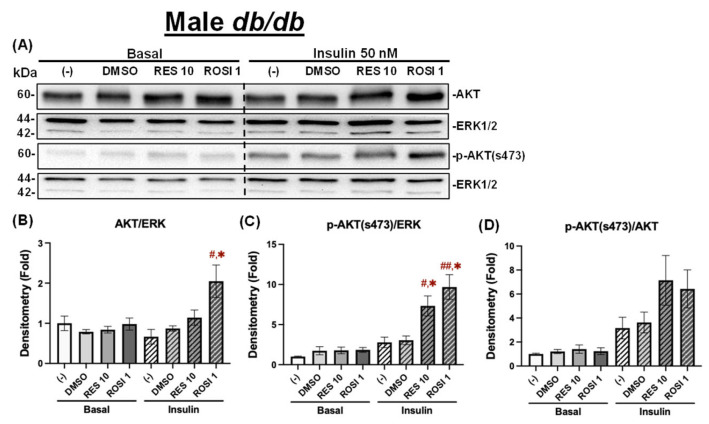
The effects of RES on insulin signaling in the differentiated ADSCs from male *db*/*db* mice. The ADSCs from male *db*/*db* mice were differentiated for 7 days in the presence or absence of RES (10 µM), ROSI (a positive control, 1 μM), or DMSO, followed by overnight starvation in DMEM/high glucose without FBS. Cells were then stimulated with or without insulin (50 nM) for 20 min. Protein lysates were collected for Western blot analysis. (**A**) Total AKT, *p*-AKT (ser473), and their respective loading control ERK1/2 are shown. The left panel represents the basal condition (−Insulin), and the right panel shows the insulin-stimulated condition (+Insulin). Quantifications of total AKT/ERK1/2 (**B**), *p*-AKT/ERK1/2 (**C**), and *p*-AKT/total AKT (**D**) are presented as fold changes relative to the (−) group under basal conditions. Data are shown as mean ± SEM (*n* = 3). One-way ANOVA was used for statistical analysis. ^#^ and ^##^ indicate *p* < 0.05 and *p* < 0.01 compared to the (−) group. * indicates *p* < 0.05 compared to the DMSO group.

Similarly To the differentiated male WT ADSCs, ROSI, but not RES, significantly increased total AKT protein levels under insulin-stimulated conditions in the differentiated male *db*/*db* ADSCs (Figure 10A,B). Similarly, both RES and ROSI significantly increased *p*-AKT (Ser473) levels compared to the (−) and DMSO controls (Figure 10C). However, no significant differences were observed in *p*-AKT/AKT ratios across groups, although both RES and ROSI showed a non-significant trend toward increased *p*-AKT/AKT ratios compared to both controls (Figure 10D).

**Figure 11:**
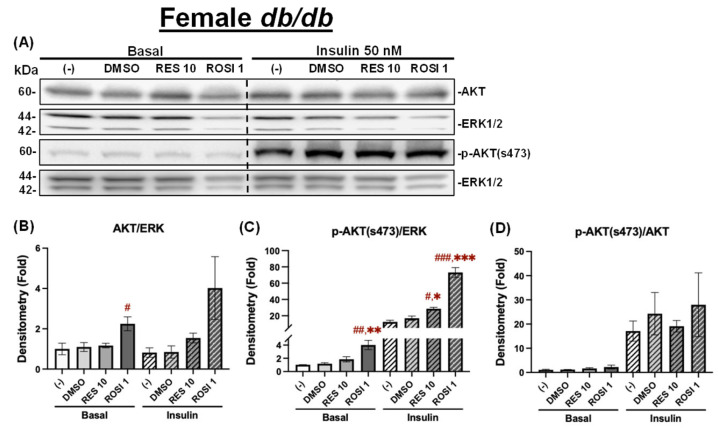
The effects of RES on insulin signaling in the differentiated ADSCs from female *db*/*db* mice. The ADSCs from the female *db*/*db* mice were differentiated for 7 days in the presence or absence of RES (10 µM), ROSI (a positive control, 1 μM), or DMSO, followed by overnight starvation in DMEM/high glucose without FBS. Cells were then stimulated with or without insulin (50 nM) for 20 min, and protein lysates were collected for Western blot analysis. (**A**) Total AKT, *p*-AKT (ser473), and their respective loading control ERK1/2 are shown. The left panel corresponds to the basal condition (−Insulin), and the right panel to the insulin-stimulated condition (+Insulin). Quantifications of total AKT/ERK1/2 (**B**), *p*-AKT/ERK1/2 (**C**), and *p*-AKT/total AKT (**D**) are presented as fold changes relative to the (−) group under basal conditions. Data are shown as mean ± SEM (*n* = 3). One-way ANOVA was used for statistical analysis. ^#^, ^##^, and ^###^ indicate *p* < 0.05, *p* < 0.01, and *p* < 0.001, respectively, compared to the (−) group. *, **, and *** indicate *p* < 0.05, *p* < 0.01, and *p* < 0.001, respectively, compared to the DMSO group.

In contrast, ROSI seemed to increase total AKT levels under both basal and insulin-stimulated conditions, only reaching significance under basal conditions compared to the (−) control, while RES did not have any effects on total AKT under both conditions in the differentiated female *db*/*db* ADSCs (Figure 11A,B). While ROSI also significantly elevated *p*-AKT (Ser473) levels under both basal and insulin-stimulated conditions compared to both the (−) and DMSO controls, RES only increased *p*-AKT (Ser473) levels under insulin-stimulated conditions (Figure 11C). However, no significant differences were observed in the *p*-AKT/AKT ratios with ROSI or RES compared to both controls under either basal or insulin-stimulated conditions in the differentiated female *db*/*db* ADSCs (Figure 11D).

### 3.6. Influences of Sex and Diabetes Status on Improved Insulin Signaling by RES

**Figure 12:**
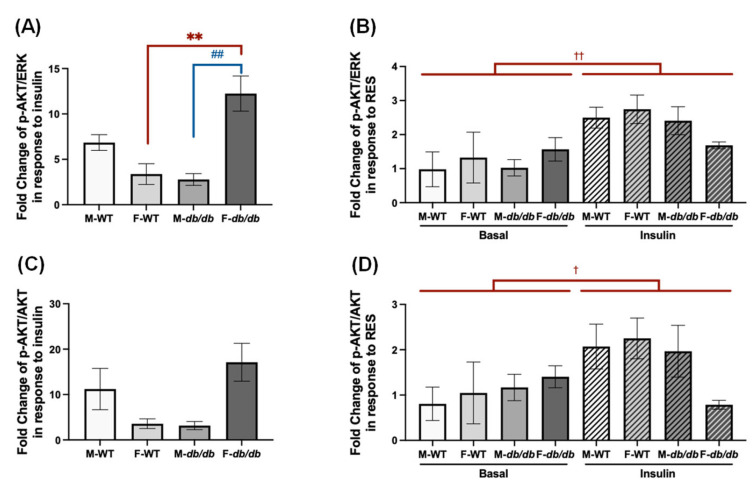
Fold changes in insulin sensitivity markers following insulin and RES treatment in the differentiated ADSCs from male and female WT and *db*/*db* mice. (**A**) Fold changes in *p*-AKT/ERK and (**C**) *p*-AKT/AKT in response to insulin were calculated for all groups. (**B**) Fold changes in *p*-AKT/ERK and (**D**) *p*-AKT/AKT in response to RES relative to the DMSO group under basal and insulin-stimulated conditions were calculated. Data are shown as mean ± SEM (*n* = 3). One-way ANOVA was used for statistical analysis in (A,C), and two-way ANOVA was used in (B,D). ^##^ indicates *p* < 0.01 for comparisons between males and females, while ** indicates *p* < 0.01 for comparisons between WT and *db*/*db* mice. ^†^ and ^††^ indicate *p* < 0.05 and *p* < 0.01 for comparisons between basal and insulin conditions.

To understand the impact of genotype and sex on the differentiated ADSCs’ response to insulin and RES, we calculated fold changes in *p*-AKT/ERK and *p*-AKT/AKT in response to insulin (Figure 12A,C) and to RES relative to DMSO under basal and insulin-stimulated conditions (Figure 12B,D) for the four groups of differentiated ADSCs. Insulin induced significantly higher *p*-AKT levels in the differentiated female *db*/*db* ADSCs than in differentiated female WT and male *db*/*db* ADSCs (Figure 12A). However, there were no significant differences in the *p*-AKT/AKT ratios in response to insulin among all four groups (Figure 12C). Although RES induced higher levels of *p*-AKT and higher *p*-AKT/AKT ratios under insulin-stimulated conditions in all groups of differentiated ADSCs, there were no significant differences among groups in *p*-AKT or *p*-AKT/AKT in response to RES under either basal or insulin-stimulated conditions (Figure 12D).

## 4. Discussion

This study demonstrates that RES at a physiologically achievable dose of 10 μM promotes the browning of adipocytes and modulates insulin signaling in beige adipocytes differentiated from the subcutaneous ADSCs of both WT and diabetic (*db/db*) mice. To our knowledge, this is the first study that has directly compared the effects of RES on browning and insulin signaling in differentiated ADSCs derived from diabetic mice compared with their WT controls, as well as in females compared to their male counterparts. These results are further summarized in Supplementary Tables S1–S3. Our results demonstrate that sex and diabetic status affect the responses of differentiated ADSCs to physiological stimulations, such as the β-adrenergic agonist isoproterenol (ISO) and insulin, as well as to the dietary bioactive compound RES.

Adrenergic activation, primarily through the sympathetic nervous system, plays a crucial role in thermogenesis and energy expenditure as heat in brown and brown-like adipocytes. ISO, a nonselective β-adrenergic agonist, induces thermogenic activation by upregulating *Pgc1α* and *Ucp1* mRNA expression in all four groups of differentiated ADSCs. Higher ISO-stimulated *Pgc1α* mRNA induction appeared in the differentiated *db*/*db* ADSCs of both sexes. However, these differences were not statistically significant compared to their WT counterparts (Figure 2A). Moreover, the differentiated ADSCs from female *db*/*db* mice showed significantly higher ISO-stimulated *Ucp1* mRNA induction compared to female WT controls or their male *db*/*db* counterparts (Figure 2C). These results suggest that cellular adrenergic activation pathways in differentiated ADSCs from *db*/*db* mice are functional and may be upregulated to some extent, compensating for the genetic defects.

Regarding sex differences in browning response, it is found that the differentiated ADSCs from female WT mice had a similar browning response to RES compared to those from male WT mice (Figure 2B,D and Figure 7). And it is the cells from female *db*/*db* mice that showed the highest response to ISO and insulin (Figure 2A,C and Figure 12). The observed sex differences in ISO-induced *Ucp1* mRNA upregulation may not be due to sex-specific differences in adrenergic receptor expression, since there were no significant differences in β3 adrenergic receptor protein expression in the inguinal subcutaneous fat between male and female WT and *db*/*db* mice (Supplementary Figure S1), contrary to what was reported for endothelial β-adrenoceptors [30]. RES is known to induce browning of white adipocytes differentiated from adipose-derived stromal cells [15]. The browning effects may be attributed to activation of AMP-activated protein kinase (AMPK), peroxisome proliferator-activated receptor gamma coactivator 1 alpha (PGC1α), and proliferator-activated receptor gamma (PPARγ) [13,15,16]. However, it is unknown whether these browning effects are modulated by sex and diabetic status of adipose-derived stromal cells. To our surprise, RES induced similar *Ucp1* mRNA upregulation under both basal and ISO-stimulated conditions across all groups of differentiated ADSCs (Figure 2D). In contrast, the induction of *Pgc1α* mRNA expression by RES, particularly under ISO-stimulated conditions, was more robust in the differentiated ADSCs from the *db*/*db* mice compared to WT mice in both sexes, and female *db*/*db* cells had a greater response to RES compared to the male *db*/*db* cells (Figure 2B). These results suggest that differentiated *db*/*db* ADSCs can respond to the browning effects of RES (even to a higher degree). Our findings not only align with a previous report demonstrating RES’s browning effects on the differentiated white adipose stromal cells from female wildtype mice [15], but also extend these observations to white adipose stromal cells from diabetic *db*/*db* mice of both sexes. Interestingly, in one study [16], only newborn male, but not female, mice showed improved metabolism and browning of inguinal white adipose tissue when orally exposed to RES from day 2 to 20 of life, and when exposed to a high-fat diet from day 90 for 10 weeks. In a follow-up study [17], RES induced *Ucp1* mRNA expression in the differentiated adipocytes derived from stromal cells of inguinal white adipose tissue from male, but not female, mice neonatally exposed to RES. These differences are likely due to differences in strains of mice (NMRI vs. C57BL6J) and RES treatment protocols (ex vivo vs. in vitro). Therefore, more studies are needed in the future to confirm the sex differences in RES’s effects on browning and the underlying mechanisms.

To correlate thermogenic gene expression, both mitochondrial respiration and uncoupling in response to RES were further examined in the four groups of differentiated ADSCs. Although there were no significant differences in RES-induced changes in proton leak (Figure 7A), there were significant differences in RES-induced changes in coupling efficiency among the four groups of differentiated ADSCs (Figure 7B), as differentiated male *db*/*db* ADSCs showed significantly lower coupling efficiency (i.e., higher uncoupling) in response to RES than male WT cells and female *db*/*db* cells (Figure 7B). The fact that RES induced similar changes in *Ucp1* mRNA expression but significant differences in mitochondrial coupling efficiency in the four groups of differentiated ADSCs suggests that (i) RES may have differential effects on ATP production among the four groups of differentiated ADSCs, and (ii) RES may have differential effects on the *Ucp1* gene post-transcription, post-translation, and/or post-translational modification. It is known that the functionality of the UCP1 protein is determined not only by *Ucp1* gene expression but also by its post-translational modifications, which are controlled by fatty acid presence, purine nucleotides, and the cellular redox state [31,32,33]. Hence, future studies are needed to explore the mechanisms underlying the differential effects of RES in reducing mitochondrial uncoupling in the four groups of differentiated ADSCs. Moreover, the results of this study underscore the importance of assessing mitochondrial uncoupling in conjunction with thermogenic gene expression when studying the browning effects in differentiated ADSCs from diabetic versus WT animals, and in males versus females.

Insulin signaling was further studied in the differentiated ADSCs with or without RES treatment. Although insulin-induced *p*-AKT levels (i.e., *p*-AKT/ERK) varied (Figure 12A), insulin-induced *p*-AKT/AKT ratios did not differ among the four differentiated ADSCs (Figure 12C), indicating that although insulin may have differential effects on total AKT levels, differentiated ADSCs respond to insulin with a similar degree of AKT phosphorylation, regardless of sex and diabetic status. We used insulin at 50 nM, a supraphysiological concentration compared to random serum insulin levels (~20 ng/mL or ~3.4 nM, our unpublished data), in the *db*/*db* mice. These results suggest that differentiated *db*/*db* ADSCs respond to supraphysiological levels of insulin in a similar manner to differentiated WT ADSCs.

In addition to influencing mitochondrial uncoupling and energy expenditure, RES showed similar fold increases in *p*-AKT (Figure 12B) and *p*-AKT/AKT (Figure 12D) under insulin-stimulated conditions in the differentiated ADSCs, as there were no differences in RES-induced *p*-AKT and *p*-AKT/AKT ratios under insulin-stimulated conditions among the four groups of differentiated ADSCs (Figure 12B,D). These results suggest that under the tested conditions, RES has similar beneficial effects in enhancing insulin signaling in differentiated ADSCs, regardless of sex or diabetic condition.

Our study has several limitations. First, the experiments were performed in vitro with differentiated ADSCs, which does not capture the full complexity of adipose tissue in vivo, including the interplay of systemic hormones, nervous connections, and immune components. In addition, while we assessed gene expression and mitochondrial respiration, we did not quantify other functional outcomes, such as glucose uptake and fatty acid oxidation. Furthermore, only one dose of RES and the stromal cells from only one fat depot were investigated. Lastly, although the *db*/*db* mouse model reflects important features of obesity and obesity-associated T2DM, further research using human cells or mouse transplantation models may be essential for establishing a browning strategy for treating human obesity and T2DM.

## 5. Conclusions

Our studies confirm the therapeutic potential of RES as a potent bioactive phytonutrient that promotes browning of white adipocytes, accompanied by increased mitochondrial uncoupling, in differentiated ADSCs. We further demonstrate that sex and diabetic status modulate the effects of RES on browning, but not the improvement of insulin signaling. Future work should focus on validating these findings in vivo, elucidating the molecular pathways underlying sex- and genotype-related differences, and translating the RES-based browning approach into a personalized treatment for human obesity and T2DM.

## Data Availability

The original contributions presented in this study are included in the article/Supplementary Materials. Further inquiries can be directed to the corresponding author.

## Supplementary Materials

The following supporting information can be downloaded at: https://www.mdpi.com/article/10.3390/nu18010019/s1, Table S1: Summary of *Ucp1* and *Pgc1* mRNA expression in response to RES in the four groups of differentiated ADSCs; Table S2: Summary of proton leak and coupling efficiency in response to RES in the four groups of differentiated ADSC; Table S3: Summary of insulin-stimulated AKT phosphorylation in response to RES in the four groups of differentiated ADSCs; Figure S1: β3-Adrenergic Receptor (ADRB3) protein expression in the inguinal subcutaneous white adipose tissue (WAT) from WT and *db/db* mice of both sexes.
