# Supplementary material for: Effects of Resveratrol on Browning and Insulin Signaling in Primary Murine Adipocytes: Modulation by Sex and Diabetic Status
Source: Nutrients. 2025 Dec 19;18(1):19. doi: 10.3390/nu18010019 (PMC12788144; doi:10.3390/nu18010019)
Supplement: Supplementary file 1 [file nutrients-18-00019-s001.zip › nutrients-4018510-supplementary.pdf]

Supplemental data

Effects of Resveratrol on Browning and Insulin Signaling in Primary Murine Adipocytes: Modulation by Sex and Diabetic Status

By Xinyun Xu [1,#], Haoyin Wu [1], Jiangang Chen [2], Shu Wang [3] and Ling Zhao [1,*]

Table S1. Summary of *Ucp1* and *Pgc1* mRNA expression in response to RES in the four groups of differentiated ADSCs.

Ucp1	Basal condition	ISO-stimulated	*Pgc1a*	Basal condition	ISO-stimulated
**Male WT**	# vs. (-)	# vs. (-)		ns vs. (-)	ns vs. (-)
	ns vs. DMSO	* vs. DMSO		ns vs. DMSO	ns vs. DMSO
**Female WT**	# vs. (-)	## vs. (-)		ns vs. (-)	## vs. (-)
	ns vs. DMSO	** vs. DMSO		ns vs. DMSO	* vs. DMSO
**Male *db/db***	### vs. (-)	### vs. (-)		# vs. (-)	### vs. (-)
	*** vs. DMSO	*** vs. DMSO		** vs. DMSO	*** vs. DMSO
**Female *db/db***	# vs. (-)	## vs. (-)		ns vs. (-)	## vs. (-)
	* vs. DMSO	** vs. DMSO		* vs. DMSO	** vs. DMSO

ns, not significantly different. #, ##, ###, indicate $p < 0.05$, $p < 0.01$, and $p < 0.001$, respectively, compared to the negative control (-) group.

*,**,***, indicate $p < 0.05$, $p < 0.01$, and $p < 0.001$, respectively, compared to the DMSO group.

Table S2. Summary of proton leak and coupling efficiency in response to RES in the four groups of differentiated ADSCs.

	Proton Leak	Coupling Efficiency
**Male WT**	ns vs. (-)	# vs.(-)
	ns vs. DMSO	ns vs. DMSO
**Female WT**	# vs. (-)	# vs. (-)
	ns vs. DMSO	* vs. DMSO
**Male *db/db***	# vs. (-)	### vs. (-)
	* vs. DMSO	** vs. DMSO
**Female *db/db***	## vs. (-)	### vs. (-)
	ns vs. DMSO	*** vs. DMSO

ns, not significantly different. #, ##, ###, indicate $p < 0.05$, $p < 0.01$, and $p < 0.001$, respectively, compared to the negative control (-) group.

*,**,***, indicate $p < 0.05$, $p < 0.01$, and $p < 0.001$, respectively, compared to the DMSO group.

Table S3. Summary of insulin-stimulated AKT phosphorylation in response to RES in the four groups of differentiated ADSCs.

	**p-AKT/ERK**	**p-AKT/AKT**
**Male WT**	## vs. (-)	ns vs.(-)
	∗∗ vs. DMSO	∗ vs. DMSO
**Female WT**	# vs. (-)	# vs. (-)
	∗ vs. DMSO	∗ vs. DMSO
**Male *db/db***	# vs. (-)	ns vs. (-)
	∗ vs. DMSO	ns vs. DMSO
**Female *db/db***	# vs. (-)	ns vs. (-)
	∗ vs. DMSO	ns vs. DMSO

ns, not significantly different. #, ##, indicate $p < 0.05$ and $p < 0.01$, respectively, compared to the negative control (-) group.

∗,∗∗, indicate $p < 0.05$ and $p < 0.01$, respectively, compared to the DMSO group.

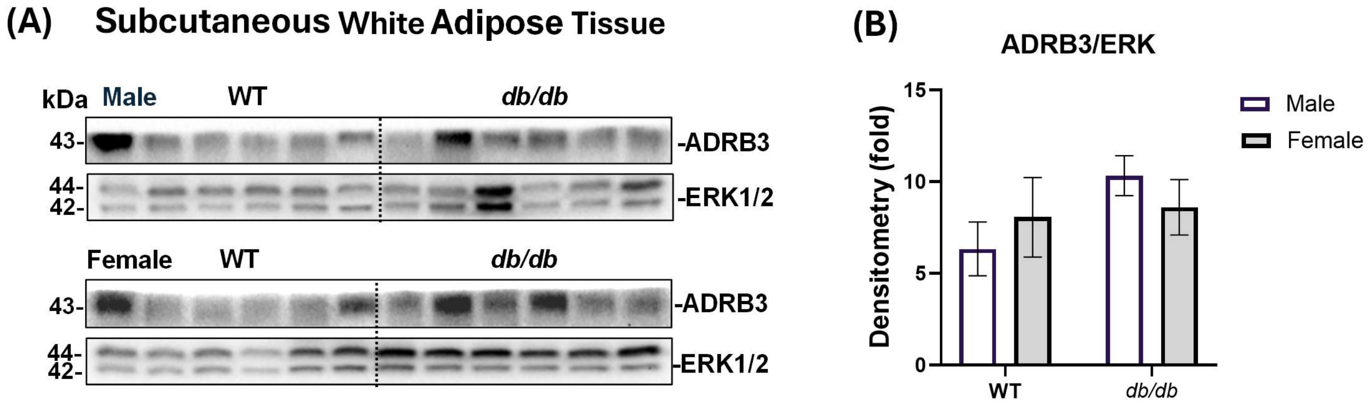

Figure S1. β3-Adrenergic Receptor (ADRB3) protein expression in the inguinal subcutaneous white adipose tissue (WAT) from WT and *db/db* mice of both sexes.

(A) Protein expression of ADRB3 and the loading control ERK1/2 in the inguinal subcutaneous WAT of WT and *db/db* male (top) and female mice (bottom). (B) Fold changes of densitometric quantifications of ADRB3 and ERK1/2 protein expression. Densitometry was quantified using ImageJ software. Data = mean ± SEM (n = 6). No statistical significance was detected by two-way ANOVA followed by Sidak's multiple comparison tests. ADRB3 antibody was purchased from Sigma-Aldrich (Product # SAB4500584) and ERK1/2 antibody was purchased from Cell Signaling Technologies (Cat# 4695S).
